# Access to routinely collected health data for clinical trials – review of successful data requests to UK registries

**DOI:** 10.1186/s13063-020-04329-8

**Published:** 2020-05-12

**Authors:** Sarah Lensen, Archie Macnair, Sharon B. Love, Victoria Yorke-Edwards, Nurulamin M. Noor, Meredith Martyn, Alexandra Blenkinsop, Carlos Diaz-Montana, Graham Powell, Elizabeth Williamson, James Carpenter, Matthew R. Sydes

**Affiliations:** 1 MRC Clinical Trials Unit at UCL, Health Data Research, London, WC1V 6LJ UK; 2 Department of Molecular and Clinical Pharmacology, University of Liverpool, Liverpool, L69 3BX UK; 3 London School of Hygiene and Tropical Medicine, London, WC1E 7HT UK; 4 Medical Statistics, London School of Hygiene and Tropical Medicine, London, WC1E 7HT UK

**Keywords:** Systematic review, Routinely collected health data, Registry, RCT

## Abstract

**Background:**

Clinical trials generally each collect their own data despite routinely collected health data (RCHD) increasing in quality and breadth. Our aim is to quantify UK-based randomised controlled trials (RCTs) accessing RCHD for participant data, characterise how these data are used and thereby recommend how more trials could use RCHD.

**Methods:**

We conducted a systematic review of RCTs accessing RCHD from at least one registry in the UK between 2013 and 2018 for the purposes of informing or supplementing participant data. A list of all registries holding RCHD in the UK was compiled. In cases where registries published release registers, these were searched for RCTs accessing RCHD. Where no release register was available, registries were contacted to request a list of RCTs. For each identified RCT, information was collected from all publicly available sources (release registers, websites, protocol etc.). The search and data extraction were undertaken between January and May 2019.

**Results:**

We identified 160 RCTs accessing RCHD between 2013 and 2018 from a total of 22 registries; this corresponds to only a very small proportion of all UK RCTs (about 3%). RCTs accessing RCHD were generally large (median sample size 1590), commonly evaluating treatments for cancer or cardiovascular disease. Most of the included RCTs accessed RCHD from NHS Digital (68%), and the most frequently accessed datasets were mortality (76%) and hospital visits (55%). RCHD was used to inform the primary trial (82%) and long-term follow-up (57%). There was substantial variation in how RCTs used RCHD to inform participant outcome measures. A limitation was the lack of information and transparency from registries and RCTs with respect to which datasets have been accessed and for what purposes.

**Conclusions:**

In the last five years, only a small minority of UK-based RCTs have accessed RCHD to inform participant data. We ask for improved accessibility, confirmed data quality and joined-up thinking between the registries and the regulatory authorities.

**Trial registration:**

PROSPERO CRD42019123088.

## Background

Randomised controlled trials (RCTs) are the gold-standard method for evaluating health-care interventions and their results impact on policy, practice, and patient care. Substantial resources are dedicated to the collection of trial data and participant follow-up. Consequently, the costs of conducting large trials are substantial, maybe in the order of millions of pounds [1]. However, many national databases and registries collect data that map to common important health-care events such as hospital admission, cancer registration and death. Use of this routinely collected health data (RCHD) to replace or supplement traditional data capture should reduce trial costs, enabling a greater number of large, definitive trials and efficient long-term assessment of health-care interventions.

This explains why the use of RCHD in RCTs has been labelled a disruptive technology (i.e. a technology that transforms current practice) [2]. A model exemplar is the TASTE (Thrombus Aspiration in ST-Elevation Myocardial Infarction in Scandinavia) trial, which randomly assigned 7244 participants in two years within national Swedish registries, collected participant data from registries and yielded high-impact results at a small fraction of the cost of traditional RCTs (USD $300,000 or $50 per patient) [3, 4]. The UK holds a large number of rich health datasets, linkable through a unique National Health Service (NHS) number. The availability of these datasets is growing, as are the technological capabilities of processing and storing these data. In response to this, Health Data Research (HDR) UK was established with the ambition of unleashing the potential of RCHD to deliver “Better, Faster and More Efficient Trials” [5].

However, while RCHD is already being harnessed to enhance UK RCTs, anecdotal evidence suggests that substantial barriers remain. Therefore, this systematic review set out to identify and characterise RCTs accessing RCHD in the UK to inform participant data, to describe how RCTs use these data, and to prioritise issues that need to be addressed.

## Methods

We conducted a systematic review of RCTs that have accessed RCHD to inform or supplement trial data.

### Eligibility

RCHD was defined as data that are collected for “administrative and clinical purposes without specific a priori research goals” [6]. This included large, national, administrative resources (e.g. NHS Digital) and national disease and health-care audits and registries in each UK devolved nation (e.g. the National Emergency Laparotomy Audit). Hereafter, we refer to these collectively as registries. Cohort studies, biobanks, NHS Safe Havens and electronic health records held only at the point of care, such as primary care records held within general practitioner (GP) practices, were excluded.

Eligible RCTs received RCHD from a registry between 2013 and 2018. This time frame was selected to broadly align with the initiation of release registers in large national databases following the 2014 Partridge Review [7]. For each included RCT, any additional access to RCHD from another registry and any previous access of RCHD prior to 2013 were also captured.

Eligible RCTs were those that accessed RCHD to inform either baseline or outcome measure data of trial participants. For at least one outcome measure, RCHD must have been used for any combination of the following: (i) replacing conventionally collected trial data, (ii) cross-checking against existing trial data (including participant-reported data), (iii) cross-checking RCHD from different sources, (iv) triggering the trial team to further investigate a possible outcome measure or event, (v) cost-effectiveness analysis and (vi) solely methodological purposes. This was captured separately for (a) the primary reporting period of the trial (i.e. baseline data or an outcome measure within the follow-up for capturing the primary trial outcome measure) and (b) long-term follow-up.

We excluded RCTs if the RCHD was accessed only to plan or facilitate recruitment (e.g. to contact patients with an invitation of RCT enrolment) or to extrapolate results of RCTs to broader populations. The protocol for this review was registered with PROSPERO at the stage of screening and data collection (CRD42019123088, registered 20 February 2019).

### RCT identification

First, we compiled a list of registries (health-care databases, registries and audits) in the UK through internet searching, the Health Quality Improvement Program (HQIP) directory [8], contact with government and contracted organisations, and existing knowledge of UK registries (more information on registries approached in Additional file 1). Release registers were identified where possible; these are lists of all data released from a given registry, often including the purpose for which the data will be used and the specific datasets accessed. Where these were not available, registries were contacted to request a list of RCTs to which they had released RCHD.

Release registers from each source were de-duplicated prior to screening (to remove multiple instances of data releases for the same RCT from an individual registry). The resultant list was searched for eligible RCTs by filtering for entries containing one or more of the following terms: rand*, trial, RCT, study, placebo and phase. The search results were screened independently for potentially eligible RCTs by two authors. Disagreements were resolved by discussion and re-checking.

### Data collection and analysis

For each RCT identified, we sought information from within the release registers (e.g. ‘statements of purpose’), RCT websites (including privacy statements, publications, protocols, statistical analysis plans, patient information sheets and consent forms) and other available sources, including trial registration information. Publications for each RCT were searched for in major dissemination databases (e.g. MEDLINE and Google Scholar). More information about data collection is given in an Additional file 4.

Data collection included information about the RCT (e.g. disease category, recruitment and publication status, and primary outcome measure), the registry (e.g. NHS Digital), the RCHD accessed (e.g. Hospital Episode Statistics) and the way in which the data were used (e.g. linkage identifiers used and category of data use). Owing to the large number of RCTs identified, we focussed more detailed data collection of information on the datasets accessed and the way in which the data were used to RCTs accessing RCHD between 2017 and 2018. Two authors independently extracted data onto a piloted data extraction form, and any disagreements were resolved by discussion and re-checking. Data were subsequently entered into a clinical data management system (Elsevier’s MACRO [9]), and descriptive analyses were undertaken in Stata (version 15.1, [10]). Trial teams were not contacted for information or clarification. To enable a broad comparison of this cohort of RCTs with those conducted in the UK, we compared the descriptive characteristics of these RCTs with those reported in a recent cross-sectional analysis of UK Health Research Authority (HRA) approved RCTs [11].

### Patient and public involvement

No patients were involved in any component of the design, production, analysis, interpretation or writing up of the results of this review. We plan to disseminate the final results to the HDR UK Public Advisory Board and request that they disseminate the manuscript within their network as appropriate.

## Results

### Results of the search

**Fig. 1:**
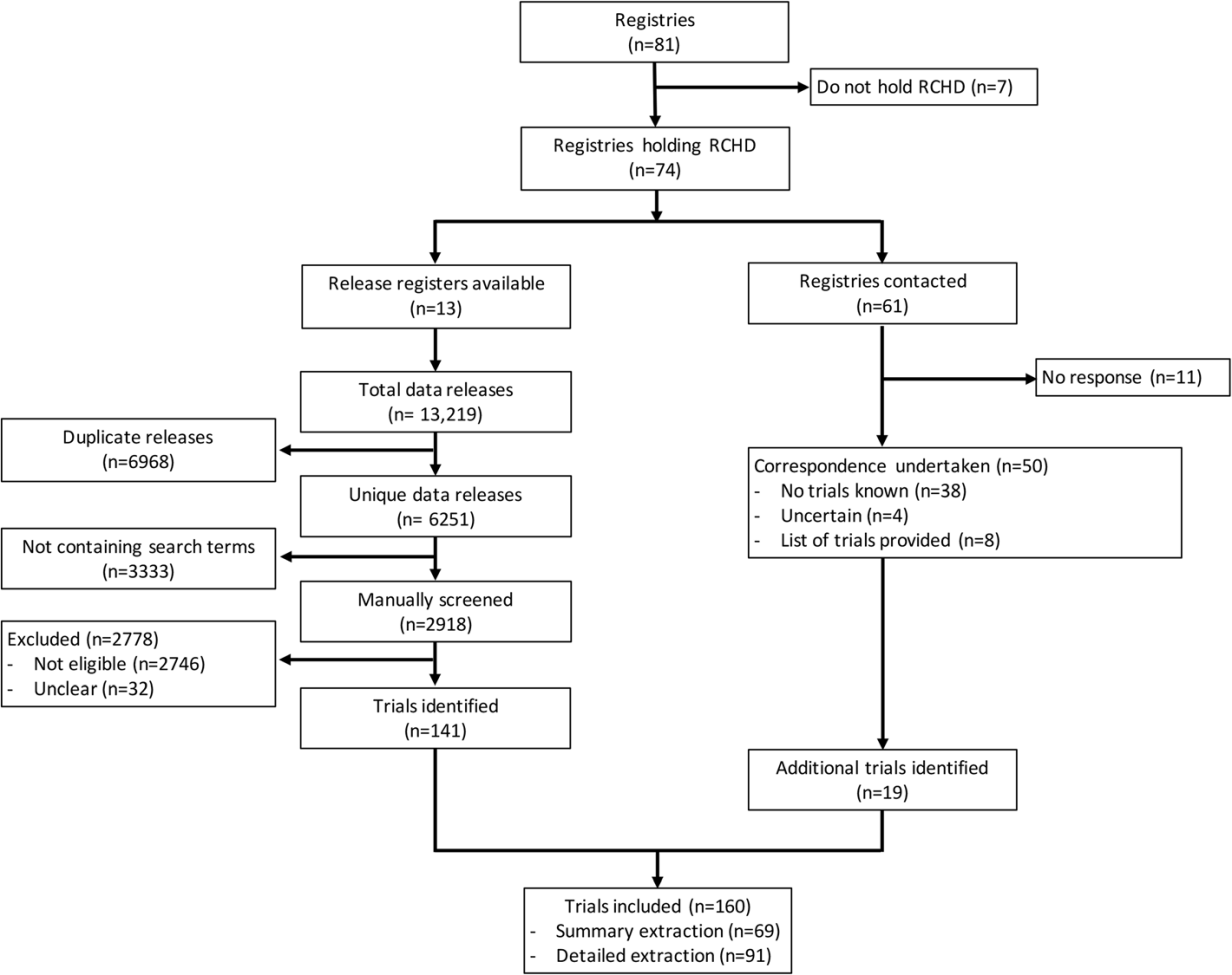
Identification of trials from registries. Each trial is counted only once. For instance, trials identified through both release register searches and notification by registries are captured only once. Of 13 registries with release registers available, 10 published comprehensive release registers and three provided a brief lists of projects receiving routinely collected health data (RCHD) on the website

The search and extraction of data were undertaken between January and May 2019; 74 UK registries holding RCHD were identified and 13 of these maintained accessible release registers (Fig. 1).

These release registers listed more than 6000 unique data releases; 2918 releases were identified in the search. These were manually screened and 141 RCTs were identified, corresponding to 2% (141/6251) of the total releases. The remaining 61 registries were contacted to request information about RCTs having accessed RCHD, resulting in a further 19 RCTs identified from eight registries. During the data extraction, we discovered one trial that had received data from one of the registries which had not otherwise provided a list of trials. This gave a total of 160 RCTs accessing RCHD from 22 registries between 2013 and 2018 (Fig. 1). Although all RCTs had accessed RCHD between 2013 and 2018, they were conducted in varying time periods; recruitment start dates ranged from 1979 to 2018. Detailed data collection, for trials accessing data in 2017–18, involved 91 out of 160 trials.

### RCT characteristics

The 160 included RCTs were generally large (median sample size 1590, range 41–6,000,000), although 11% (17/160) described themselves as pilot or feasibility trials (Table 1). The majority (85%, 136/160) were individually randomised trials, and 15% (24/160) were cluster-randomised. The most common disease categories were cancer (29%, 47/160) and cardiovascular disease (29%, 46/160), and the most common primary outcome measure was survival/death (45%, 72/160). Only 20% (32/160) of the RCTs were international, recruiting at additional sites outside of the UK. A small number of RCTs had publications available which included outcome measures informed by access to RCHD. Of these, 83% (29/35) had one or more results published in a high-profile medical journal.

**Table 1:** Trial characteristics

Trial characteristic	Summary extraction: 2013–16, n (%) ***n*** = 69	Detailed extraction: 2017–18, n (%) ***n*** = 91	Total, n (%) ***n*** = 160
**Purpose**			
Screening	6 (9%)	10 (11%)	16 (10%)
Treatment	51 (74%)	65 (71%)	116 (73%)
Primary prevention	12 (17%)	16 (18%)	28 (18%)
**Feasibility/Pilot trial**			
Yes	6 (9%)	11 (12%)	17 (11%)
No	63 (91%)	80 (88%)	143 (89%)
**Recruitment setting**			
Primary care	23 (33%)	18 (20%)	41 (26%)
Secondary care	46 (67%)	73 (80%)	119 (74%)
**Disease category**			
Cancer	17 (25%)	30 (33%)	47 (29%)
Cardiovascular and stroke	21 (30%)	25 (27%)	46 (29%)
Pregnancy and childbirth	4 (6%)	5 (5%)	9 (6%)
Mental/neurological health	6 (9%)	6 (7%)	12 (8%)
Infection	5 (7%)	3 (3%)	8 (5%)
Endocrine and diabetes	1 (1%)	3 (3%)	4 (3%)
Inflammatory disorder	2 (3%)	3 (3%)	5 (3%)
Other	13 (19%)	16 (18%)	29 (18%)
**Intervention**			
Drug	38 (55%)	38 (42%)	76 (48%)
Surgical	5 (7%)	8 (9%)	13 (8%)
Other	26 (38%)	45 (49%)	71 (44%)
**Primary outcome**			
Survival related	33 (48%)	39 (43%)	72 (45%)
Other	36 (52%)	52 (57%)	88 (55%)
**Randomisation**			
Individual	61 (88%)	75 (82%)	136 (85%)
Cluster	8 (12%)	16 (18%)	24 (15%)
**Trial size**			
**Median**	1103	2611	1590
**Range**	41–170,432	53–6,000,000	41–6,000,000
1–500	21 (30%)	20 (22%)	41 (26%)
500–5000	31 (45%)	43 (47%)	74 (46%)
>5000	17 (25%)	25 (27%)	42 (26%)
Unclear	0 (0%)	3 (3%)	3 (2%)
**International accrual**			
Yes	13 (19%)	19 (21%)	32 (20%)
No (UK only)	56 (81%)	69 (76%)	125 (78%)
Unclear	0 (0%)	3 (3%)	3 (2%)
**Coordinated by registered clinical trials unit**			
Yes	40 (58%)	63 (69%)	103 (64%)
No	10 (14%)	12 (13%)	22 (14%)
Unclear	19 (28%)	16 (18%)	35 (22%)
**Highest profile journal (if primary report published)**			
*BMJ*	NA	2 (2%)	2 (1%)
*JAMA*	NA	6 (7%)	6 (4%)
*Lancet*	NA	16 (18%)	16 (10%)
*Lancet-specialty*	NA	2 (2%)	2 (1%)
*NEJM*	NA	3 (3%)	3 (2%)
Other	NA	6 (7%)	6 (4%)
Not yet published	NA	56 (62%)	56 (35%)
Not captured	69 (100%)	NA	69 (43%)

The majority of RCTs were clearly coordinated through a UK Clinical Research Collaboration (UKCRC)-registered clinical trials unit (CTU): 64% (103/160) were coordinated by a registered CTU, 14% (22/160) were not and 22% (35/160) were unclear) (Table 1). Of all 51 currently registered CTUs, 63% (32/51) had accessed RCHD for at least one RCT in this cohort. Of these CTUs, the median number of RCTs from this cohort was 2 (range 1–11).

RCTs accessing RCHD were more often conducted in cancer and cardiovascular populations compared with RCTs submitted for an ethical opinion via the HRA in 2015 (29% vs. 10% and 29% vs. 17%, respectively) and were more likely to recruit from primary care settings (26% vs. 5%), to be based only in the UK (78% vs. 50%) and to be cluster-randomised (15% vs. 3%). RCHD RCTs had larger sample sizes on average (median 1590 vs. 275) than those submitted to the HRA (Table 2). RCTs accessing RCHD were less likely to be feasibility/pilot studies (11% vs. 18%). We identified 160 trials accessing RCHD over a five-year period (32 trials per year), which is equivalent to about 3% (32/963) of all RCTs approved by the HRA in 2015.

**Table 2:** Comparison of RCTs accessing RCHD with trials evaluated by the HRA in 2015

	RCTs accessing RCHD (***n*** = 160)	HRA in 2015^**a**^ (***n*** = 963^**b**^)
**Recruitment setting**		
Primary care	41 (26%)	48 (5%)
Secondary care	119 (74%)	846 (95%)
Unclear/missing	0	69
**Therapeutic area**		
Cancer	47 (29%)	168 (17%)
Cardiovascular and stroke	46 (29%)	121 (13%)
Pregnancy and childbirth	9 (6%)	30 (3%)
Infection	8 (5%)	55 (6%)
Inflammatory disorder	5 (3%)	72 (7%)
**Drug trial**	76 (48%)	515 (53%)
**Randomisation**		
Individual	136 (85%)	934 (97%)
Cluster trial	24 (15%)	29 (3%)
**Feasibility/pilot**	17 (11%)	177 (18%)
**Sample size (median, range)**	1590 (41–6,000,000)	275 (6–30,000)
Unclear/missing	0	440
**Recruitment location**		
UK only	125 (78%)	450 (50%)
International trials	32 (20%)	443 (50%)
Unclear/missing	0	70

This table only includes data fields that were comparable between the two sources. Sample size targets in the UK Health Research Authority (HRA) cohort are limited to those not described as phase I/II trials. Data obtained from Clark et al. [11] (2018), including unpublished supplementary appendices [1]. *Abbreviations*: *RCHD* routinely collected health data, *RCT* randomised controlled trial

^a^Clark et al. [11].

^b^For recruitment setting and location, the unclear/missing values were omitted from the percentage calculation.

### RCHD access and use

NHS Digital was by far the most commonly accessed registry: 68% (108/160) trials accessed RCHD from NHS Digital (Table 3). The second most common was the Information Services Division in Scotland 22% (35/160). Most of the RCTs accessed RCHD from one registry (79%, 126/160); 14% (22/160) accessed data from two registries, 5% (8/160) from three registries, and 3% (4/160) from four or more. A small number of RCTs were completely embedded (i.e. participants were recruited from and followed up) in the registry (12%, 11/91).

**Table 3:** Registries and datasets accessed

Registry	Total trials***n*** = 160	Total trials (2017–2018) ***n*** = 91	Datasets accessed Total trials (2017–2018) ***n*** = 91			
			Death ***n*** = 69	Hospital visits ***n*** = 50	Cancer registration ***n*** = 29	Other ***n*** = 26
**NHS Digital**	108 (68%)	59 (65%)	58 (84%)	34 (68%)	22 (76%)	4 (15%)
**ISD-Scotland**	35 (22%)	25 (27%)	16 (23%)	13 (26%)	7 (24%)	2 (8%)
**PHE**	15 (9%)	11 (12%)	3 (4%)	6 (12%)	10 (34%)	1 (4%)
**SAIL**	9 (6%)	6 (7%)	2 (3%)	5 (10%)	2 (7%)	1 (4%)
**ICNARC**	7 (4%)	4 (4%)	1 (1%)	0 (0%)	0 (0%)	4 (15%)
**NWIS**	7 (4%)	6 (7%)	1 (1%)	6 (12%)	1 (3%)	0 (0%)
**PICANet**	6 (4%)	2 (2%)	0 (0%)	0 (0%)	0 (0%)	2 (8%)
**CPRD**	4 (3%)	1 (1%)	0 (0%)	1 (2%)	0 (0%)	1 (4%)
**NHSBT**	3 (2%)	3 (3%)	0 (0%)	0 (0%)	0 (0%)	2 (8%)
**TARN**	3 (2%)	2 (2%)	1 (1%)	1 (2%)	0 (0%)	2 (8%)
**NELA**	2 (1%)	2 (2%)	0 (0%)	0 (0%)	0 (0%)	2 (8%)
**NNRD**	2 (1%)	2 (2%)	1 (1%)	1 (2%)	0 (0%)	2 (8%)
**PHW**	2 (1%)	1 (1%)	0 (0%)	0 (0%)	0 (0%)	1 (4%)
**UKRR**	2 (1%)	2 (2%)	0 (0%)	1 (2%)	1 (3%)	0 (0%)
**ResearchOne**	2 (1%)	2 (2%)	0 (0%)	0 (0%)	0 (0%)	2 (8%)
**DOH**	1 (1%)	0 (0%)	0 (0%)	0 (0%)	0 (0%)	0 (0%)
**FFFAP**	1 (1%)	1 (1%)	0 (0%)	0 (0%)	0 (0%)	1 (4%)
**HBS**	1 (1%)	1 (1%)	0 (0%)	0 (0%)	0 (0%)	1 (4%)
**NICOR**	1 (1%)	1 (1%)	1 (1%)	0 (0%)	0 (0%)	0 (0%)
**NICR**	1 (1%)	1 (1%)	0 (0%)	0 (0%)	1 (3%)	0 (0%)
**OHCAO**	1 (1%)	1 (1%)	0 (0%)	0 (0%)	0 (0%)	1 (4%)
**UKCFR**	1 (1%)	1 (1%)	0 (0%)	0 (0%)	0 (0%)	1 (4%)

Registries accessed was captured for all 160 trials. Information about datasets accessed from these registries was captured only for those 91 accessing routinely collected health data (RCHD) between 2017 and 2018. The fields are not mutually exclusive as one trial may have accessed data from multiple registries, and multiple datasets can be accessed via a single registry. Percentages are calculated by using the column header denominators. Hospital visits includes all Hospital Episode Statistics (Outpatient, Inpatient, Accident and Emergency, and Critical Care), Patient Episode Database for Wales (PEDW), and Scottish Morbidity Records (SMR). Acronyms: Information Services Division (ISD), Public Health England (PHE), Secure Anonymised Information Linkage (SAIL), Intensive Care National Audit & Research Centre (ICNARC), NHS Wales Informatics Service (NWIS), Paediatric Intensive Care Audit Network (PICANet), Clinical Practice Research Datalink (CPRD), NHS Blood and Transplant (NHSBT), Trauma Audit and Research Network - Major Trauma Audit (TARN), National Emergency Laparotomy Audit (NELA), Neonatal Research Database (NNRD), Public Health Wales (PHW), UK Renal Registry (UKRR), Department of Health (DOH), Falls and Fragility Fractures Audit programme (FFFAP), Honest Broker Service, Northern Ireland Statistics and Research Agency (HBS), National Institute for Cardiovascular Outcomes Research (NICOR), Northern Ireland Cancer Registry (NICR), Out-of-Hospital Cardiac Arrest Outcomes (OHCAO) Registry, UK Cystic Fibrosis Registry (UKCFR)

Of the 160 RCTs, 91 had received a total of 134 data releases in the years 2017–2018 and were selected for detailed data extraction. Identifiers used for linkage were often unclear (46%, 62/134); however, when assessable, the most common fields were NHS Number (94%, 68/72), date of birth (85%, 61/72) and participant name (56%, 40/72) (an additional file shows Additional file 2: Table S2). The most common datasets accessed were mortality (76%, 69/91), hospital visits (55%, 50/91) and cancer registration (32%, 29/91) (Table 3). Almost half of the included RCTs (47%, 43/91) accessed RCHD to inform the primary trial outcome measure. Of RCTs using RCHD only for at least one outcome measure, 38% (20/52) were drug trials; 40% of RCTs (36/91) accessed RCHD for both the primary and long-term follow-up (Table 4); 21% of RCTs (19/91) accessed one or more RCHD only for long-term follow-up and 45% (41/91) accessed one or more RCHD exclusively for the primary with no obvious planned long-term follow-up.

**Table 4:** Categories describing how RCHD was used to inform or supplement participant data

Data use category	Description	Example	All trials ***N*** = 91	Primary ***N*** = 74	Long-term follow-up ***N*** = 52	Both ***N*** = 36
**1**	The RCHD alone is used as trial data, and there is no cross-checking or comparison against any other data.	One of the included RCTs was a cluster-randomised trial of GP practices. Outcome data specific to the trial were extracted at the level of the cluster (GP practice) from CPRD.	52 (57%)	33 (45%)	34 (65%)	15 (42%)
**2**	RCHD is used to cross-check against or verify known trial data, namely data already being collected on CRFs as part of the trial (arising from clinical observations and measurements).	In one case, death and cause of death of participants are captured on CRFs at each site. NHS Digital also sends the trial team quarterly reports of all new deaths. The trial team then compares the events and cause of death from both sources.	27 (30%)	24 (32%)	6 (12%)	3 (8%)
**3**	RCHD is used to cross-check against or verify self-reported trial data, namely data already being collected from participants, such as by questionnaire.	In one trial, participants are asked to complete a questionnaire every 3 months which asks whether they had any unexpected stays in hospital. This information is cross-checked against Hospital Episode Statistics data obtained from NHS Digital.	28 (31%)	22 (30%)	11 (21%)	5 (14%)
**4**	RCHD is used to alert or flag trial teams to a potential outcome/event, prompting medical note review to confirm the outcome/event. The specific outcome/events being flagged are not otherwise being captured as trial data. This may be accompanied by clinical end-point review or adjudication of events and outcomes.	In one RCT, participants were flagged in the UK Transplant Registry for notification of transplant rejection and failure. When participants were identified as having a transplant rejection or failure, study staff sought extra information from hospital records. The collated information was redacted and used for central adjudication by trained clinicians.	22 (24%)	19 (26%)	9 (17%)	6 (17%)
**5**	RCHD from one source is used to cross-check against or compared with RCHD from another source.	One of the included RCTs accessed RCHD from NHS Digital, ICNARC and OHCAO, and all had provided the same fields, such as length of stay in intensive care.	9 (10%)	7 (9%)	6 (12%)	4 (11%)
**6**	RCHD is being used for health economic analysis or cost-effectiveness purposes, rather than a clinical outcome.	In one study, Hospital Episode Statistics data were used to calculate the cost of secondary resource use within 90 days of randomisation.	25 (27%)	21 (28%)	12 (23%)	8 (22%)
**7**	RCHD is not used directly for trial purposes but to evaluate the quality of these data compared with trial data or other RCHD, or RCHD is used to generate an algorithm or equation that hopes to predict or replicate the frequency of events/outcomes.	In a breast cancer trial, cancer data from NCRAS were accessed and compared against the trial data to assess the completeness, validity and consistency of the two data sources.	11 (12%)	11 (15%)	1 (2%)	1 (3%)
**Unclear**	–	–	13 (14%)	9 (12%)	9 (17%)	5 (14%)

These categories were developed for the purpose of this review and are not mutually exclusive. For example, randomised controlled trials (RCTs) may use routinely collected health data (RCHD) for both cross-checking against existing trial data and against a second source of RCHD. Additionally, RCTs may use RCHD from multiple sources in different ways. Percentages are calculated by using the column header denominators. *Abbreviations*: *CPRD* Clinical Practice Research Datalink, *CRF* case report form, *GP* general practitioner, *ICNARC* Intensive Care National Audit & Research Centre, *NCRAS* National Cancer Registration and Analysis Service, *NHS* National Health Service, *OHCAO* Out-of-Hospital Cardiac Arrest Outcomes

Most commonly, RCHD alone was used for at least one trial outcome measure (57%, 52/91) (Table 4). One third of RCTs used RCHD for cross-checking, either of trial data (30%, 27/91) or participant-reported data (31%, 28/91). Use of RCHD to trigger case review was also common (24%, 22/91), as was use of the data to conduct cost-effectiveness analysis (27%, 25/91). Use of RCHD for methodological reasons was uncommon (12%, 11/91), as was release for comparison of two or more RCHD sources (10%, 9/91). RCTs using RCHD for long-term follow-up were more likely to use RCHD alone to inform outcome measures and less likely to conduct cross-checking against trial or participant-reported data or to use the data for methodological purposes. Overall, there was substantial variation in how trials used RCHD to inform participant outcome measures. For example, among the 74 trials using RCHD within the primary reporting period, 37 different combinations of data use were captured (an additional file shows Additional file 3: Table S3). Among the 36 RCTs using RCHD for the primary report and long-term follow-up, 56% (20/36) used the data differently for these two stages of the study for at least one outcome measure (e.g. shifting from cross-checking of trial data for the primary reporting to RCHD only during the long-term follow-up).

## Discussion

The increase in the scope, accessibility and richness of RCHD presents an unprecedented opportunity for better health research [12]. Use of RCHD for trial outcome measures may be a cost-effective means of obtaining data, limiting the burden on trial staff and participants in attending for trial visits or replying to questionnaires, especially for longer-term data collection. Use of RCHD may also minimise attrition in RCTs where datasets have national coverage, reduce issues of self-reported outcome measures which are prone to recall bias (e.g. recalling diagnoses or operations from hospital visits) and could limit ascertainment bias where the clinicians and coders are not aware of trial participation. However, are RCHD replacing case report forms in clinical trials and, if not, why not?

To the best of our knowledge, this is the first review to summarise the accessing of RCHD by randomised trials in the UK by reviewing the sources of data and the first to assess the use of these data specifically for trial outcome measure assessment. We identified 160 trials accessing RCHD to inform participant data from 22 registries in the UK between 2013 and 2018, and many (47%, 43/91) used it for the primary outcome measure. This corresponds to about 32 trials a year, which is about 3% of the trials seeking HRA approval annually [11]. Alongside this, RCTs accessing RCHD accounted for only 2% of the data releases from included registries. Since most trial patient data are captured in the hospital records, this suggests that the potential of RCHD in trials is largely untapped.

We observed considerable variation in the use of RCHD, most commonly to inform or supplement outcome measures in primary trial report and long-term follow-up. Only 52 (57%) out of 91 used RCHD alone for the collection of at least one trial outcome measure; that is, even when used, the data are duplicated from trial-specific sources – further evidence that the potential of RCHD is largely unrealised.

Only a very small proportion of UK trials appear to be successfully accessing RCHD. Our findings are consistent with anecdotal evidence that one barrier to greater access and use may be lack of awareness among trialists regarding the availability and potential utility of this information for trial follow-up. There is no national directory of registries which lists sources of RCHD available to researchers. The National Institute for Health Research (NIHR) Health Data Finder for Research contains only 18 datasets [13]. Half of the registries identified for this review confirmed that they had not provided data to RCTs and may represent an underutilised resource.

Both for us (as reviewers) and for trialists, the lack of a comprehensive list of RCHD registries and the data they hold is a challenge. Furthermore, the majority of registries we identified did not maintain a register of approved data releases. A number of release registers had brief information (e.g. only application titles), and some registries were unable to advise whether their RCHD had been released for this purpose. Therefore, our search may have missed eligible trials. For trialists, this makes it more difficult to keep abreast of how these data may be used, hindering the uptake of RCHD by the community. A further barrier is that many publications about the included trials which were expected to include RCHD made no mention of it. So it was often not clear from publically available sources exactly how RCHD would be used with a trial. (Note that we deliberately did not contact trial teams for information or clarification, as our aim was to assess information that was publically available.) The forthcoming CONSORT extension for RCTs using cohorts and routinely collected health data will cover trials accessing electronic health records and should help to improve transparency in reporting [14] and enable the community to keep abreast of developments.

Other recent reviews in this area have summarised characteristics of trials in other settings, including those using these data for at least one trial outcome measure [15, 16] and for the long-term extension of completed trials [17, 18]. These reviews identified similar types of trials accessing RCHD in terms of trial characteristics. However, owing to the traditional literature searches employed by these reviews, they identified only a handful of the UK trials identified here; by reviewing release registers rather than publications, we found that more trials are receiving data than are mentioning it in their publications.

Reliance on data provision from registries raises unpredictable, and potentially extremely time-consuming, challenges relating to data access and retention. For example, changes to registry names can render participant consent invalid if it no longer references the correct provider name. Individuals at registry organisations are also known to have provided contrary information on specific consent form wording [19]. Many researchers report long delays in the application process, impacting on timely data collection and trial completion, and there were reports that RCTs were unable to publish trial results because of issues with data access [20]; in one RCT, failure to gain access to mortality data necessitated a change to the primary trial outcome measure [21]. Cancer registration data, collected by Public Health England, have previously been available through NHS Digital; however, provision of these data stopped for a period of more than two years. Such unscheduled lapses in data availability introduce substantial risk for RCTs relying on these cancer registration notifications through this route. One RCT reported failure by the registry to update flagging of new patients as recruitment continued: the trial team received death information only for the initial half of their cohort [22]. Additionally, many registries do not permit ongoing retention or onward sharing of the datasets, creating conflict with key trial processes such as data archiving, data sharing and individual participant data meta-analysis [19, 23].

The administrative nature of some RCHD sources, the external coding and validation processes employed, and lack of oversight and visibility of data collection, processing, and audit trails raise concerning implications for Good Clinical Practice (GCP) adherence [24]. The data used in clinical trials have to be the same as the source data to be GCP-compliant. There are accounts of data quality issues from RCHD, even for clearly objective outcome measures such as death [25–27], although cardiovascular outcomes seem more promising [28]. A standardised, systematic approach to data quality assessment, ideally as a coordinated series of multi-RCT studies-within-a-trial (SWATs), would provide empirical evidence of the quality of RCHD and traditional trial data. Registry processes for data collection and editing would also need to be assessed.

The timeliness of RCHD is key. While primary care data (e.g. held by Clinical Practice Research Datalink) can be extracted easily from multiple GP practices across software systems (Vision or EMIS), provision of secondary care data such as Hospital Episode Statistics generally has delayed capture and is received in batch files every month or quarter. Certainly, these data cannot be relied upon for the timely reporting of serious adverse events (e.g. requiring hospital admission).

## Conclusion

Only a tiny percentage of UK-based RCTs have accessed RCHD in the last five years to inform participant data, and few of these are exclusively relying on RCHD, although most patient data are captured by hospital systems. Furthermore, while most RCTs appear to be using similar datasets from a small number of registries, the way in which the RCHD is used to inform or supplement trial data appeared to vary substantially. Barriers to lack of utilisation include access to data and fitness of RCHD for research purposes.

Our review supports concerns that exploiting the potential of RCHD in trials is hindered (Table 5). Targeting resources to develop robust solutions to overcome these hurdles and enable a step change for clinical trials is urgently needed so that UK trials can fully harness the power of RCHD to conduct more efficient RCTs.

**Table 5:** Barriers to use of routinely collected health data and potential solutions

Barrier	Example/explanation	Potential solution
Lack of comprehensive list of RCHD sources and the data they hold	There is no one point where you can find out about all sources of RCHD.	A searchable database – HDR UK or NHS Digital is responsible for update.
Clear terminology to describe data from registries	The source of the trial data is not always mentioned in trial publications.	Publication of consensus terminology and a description of the way in which RCHD can be used.
Publications of trials using RCHD did not make mention of it.	In methods section, details of the sources of RCHD should be stated.	Soon-to-be-released EHR CONSORT extension for routinely collected health data should improve this.
Lack of awareness by trialists of the availability and utility of RCHD	Shown by a small percentage of trials using RCHD	More publicity on available RCHD and the use of RCHD
Poor accessibility of data	Registry name change invalidating consent Long delays of several years in application process RCHD not provided despite agreement	Streamline the RCHD application process and render it efficient.
Poor data retention and no possibility of onward sharing	Time limit to keeping the data is shorter than the data storage time limit for the trial. Data sharing is often necessary to achieve funding for a trial.	RCHD needs to align with trial data retention rules and data-sharing requirements.
Data quality and timeliness	Trial data are monitored and checked and a lot of registry data are not.	Registry data need a validation process to ensure that their RCHD can be used as a verifiable GCP-compliant data source. A comparison of trial and registry data in several trials, facilitated by a SWAT, is required to educate all about the accuracy and completeness of registry data.
Regulator ready RCHD	Trial data require underlying source data whilst registry data are not source data and do not often have checked underlying source data.	Regulators and registries need to agree a solution to underlying source data.

*Abbreviations*: *EHR CONSORT* electronic health record Consolidated Standards of Reporting Trials, *GCP* Good Clinical Practice, *HDR UK* Health Data Research UK, *NHS* National Health Service, *RCHD* routinely collected health data, *RCT* randomised controlled trial, *SWAT* study-within-a-trial

## Supplementary information

**Additional file 1: Table S1.** Registries searched and approached.

**Additional file 2: Table S2.** Linkage identifier combinations by frequency of use (detailed extraction 2017–2018).

**Additional file 3: Table S3.** Data use combinations by frequency of use, among trials using data for the primary report (*n* = 74).

**Additional file 4: Appendix 1.** Additional information about data collection.

## Data Availability

All of the information is publicly available. The dataset and technical appendices are available upon request as per the controlled access approach of the Medical Research Council CTU at University College London. Please contact the corresponding author for more information.

## Abbreviations

CTU: Clinical trials unit
GCP: Good Clinical Practice
GP: General practitioner
HDR UK: Health Data Research UK
HRA: Health Research Authority
NHS: National Health Service
NIHR: National Institute for Health Research
RCHD: Routinely collected health data
RCT: Randomised controlled trial
