# Supplementary material for: Access to routinely collected health data for clinical trials – review of successful data requests to UK registries
Source: Trials. 2020 May 12;21:398. doi: 10.1186/s13063-020-04329-8 (PMC7218527; doi:10.1186/s13063-020-04329-8)
Supplement: Supplementary file 1 — Additional file 1: Table S1. Registries searched and approached. [file 13063_2020_4329_MOESM1_ESM.docx]

**Table S1. Registries searched and approached**

| **Registry (potential source of RCHD)**  **(n=74)** | **Release register available (n=13)** | **Correspondence if release register unavailable (n=61)** | | | |
| --- | --- | --- | --- | --- | --- |
|  |  | **List of trials provided (n=8)** | **No trials known (n=38)** | **Uncertain (n=4)** | **No response (n=11)** |
| Bowel Screening Wales |  |  |  | **x** |  |
| British Association of Urological Surgeons |  |  |  |  | **x** |
| British Thoracic Society, multiple audits |  |  | **x** |  |  |
| Clinical Practice Research Datalink (CPRD) | **x ^1^** |  |  |  |  |
| CORECT-R, multiple datasets |  |  | **x** |  |  |
| Department of Health (DOH) |  |  |  | **x** |  |
| Endocrine and Thyroid National Audit |  |  |  |  | **x** |
| Falls and Fragility Fractures Audit programme (FFFAP) |  | **x** |  |  |  |
| Head and Neck Cancer Audit |  |  | **x** |  |  |
| Honest Broker Service, Northern Ireland Statistics and Research Agency (HBS) | **x ^2^** |  | **x** |  |  |
| Inflammatory Bowel Disease Registry |  |  | **x** |  |  |
| Information Services Division, Scotland (ISD-Scotland) ^6^  <https://www.isdscotland.org/> | **x ^1^** |  |  |  |  |
| Intensive Care National Audit & Research Centre (ICNARC), multiple audits | **X^3^** |  |  |  |  |
| Investigation and Detection of urological Neoplasia in patients referred with suspected Urinary Tract Cancer (IDENTIFY) |  |  | **x** |  |  |
| Learning Disabilities Mortality Review Programme (LeDeR) |  |  | **x** |  |  |
| Mothers and Babies: Reducing Risk through Audits and Confidential Enquiries (MMBRACE-UK) |  |  | **x** |  |  |
| National Asthma and Chronic Obstructive Pulmonary Disease (COPD) Audit Programme (NACAP) |  |  | **x** |  |  |
| National Audit of Breast Cancer in Older People (NABCOP) |  |  | **x** |  |  |
| National Audit of Cardiac Rehabilitation |  |  | **x** |  |  |
| National Audit of Care at the End of Life (NACEL) |  |  | **x** |  |  |
| National Audit of Dementia |  |  | **x** |  |  |
| National Audit of Intermediate Care (NAIC) |  |  | **x** |  |  |
| National Audit of Seizures and Epilepsies in Children and Young People (Epilepsy12) |  |  |  |  | **x** |
| National Audit of Seizure management in Hospitals (NASH) |  |  |  |  | **x** |
| National Audit of Small Bowel Obstruction (NASBO) |  |  |  |  | **x** |
| National Audit Project of the Royal College of Anaesthetists |  |  | **x** |  |  |
| National Bariatric Surgery Registry (NBSR) |  |  | **x** |  |  |
| National Cancer Diagnosis Audit (NCDA) |  |  | **x** |  |  |
| National Cardiac Audit Programme (NCAP) |  |  |  |  | **x** |
| National Child Mortality Database |  |  | **x** |  |  |
| National Clinical Audit of Anxiety and Depression (NCAAD) |  |  | **x** |  |  |
| National Clinical Audit of Psychosis |  |  | **x** |  |  |
| National Clinical Audit of Specialist Rehabilitation for Patients with Complex Needs following Major Injury (NCASRI) |  |  |  |  | **x** |
| National Confidential Enquiry into Patient Outcome and Death (NCEPOD) |  |  | **x** |  |  |
| National Confidential Inquiry into Suicide and Safety in Mental Health (NCISH) |  |  | **x** |  |  |
| National Early Inflammatory Arthritis Audit (NEIAA) |  |  | **x** |  |  |
| National Emergency Laparotomy Audit (NELA) | **x** | **x** |  |  |  |
| National Institute for Cardiovascular Outcomes Research (NICOR) |  |  | **x ^2^** |  |  |
| National Joint Registry | **x ^4^** |  |  |  |  |
| National Lung Cancer Audit (NLCA) |  |  | **x** |  |  |
| National Maternity and Perinatal Audit (NMPA) |  |  | **x** |  |  |
| National Ophthalmology Audit (NOD) |  |  | **x** |  |  |
| National Paediatric Diabetes Audit (NPDA) |  |  | **x** |  |  |
| National Prostate Cancer Audit |  |  | **x** |  |  |
| National Vascular Registry |  |  | **x** |  |  |
| Neonatal Research Database (NNRD)/ Neonatal Intensive and Special Care (NNAP) | **x** | **x** |  |  |  |
| Neurosurgical National Audit Programme (NNAP) |  |  | **x** |  |  |
| NHS Blood and Transplant (NHSBT) |  | **x** |  |  |  |
| NHS Digital^6^  <https://digital.nhs.uk/> | **X^5^** |  |  |  |  |
| NHS Wales Informatics Service (NWIS) |  | **x** |  |  |  |
| Northern Ireland Cancer Registry (NICR) |  |  | **x ^2^** |  |  |
| Northern Ireland Registry of Self-harm |  |  | **x** |  |  |
| NorthWest EHealth (NWEH) |  |  |  | **x** |  |
| Office for National Statistics | **x** |  |  |  |  |
| Out-of-Hospital Cardiac Arrest Outcomes (OHCAO) Registry |  | **x** |  |  |  |
| Paediatric Intensive Care Audit Network  (PICANet) | **x ^3^** | **x** |  |  |  |
| Perioperative Quality Improvement Programme |  |  | **x** |  |  |
| Prescribing Observatory for Mental Health (POMH-UK) |  |  | **x** |  |  |
| Progressive multifocal leukoencephalopathy following Natalizumab |  |  |  |  | **x** |
| Public Health England (PHE) ^6^  <https://www.gov.uk/government/organisations/public-health-england> | **x ^5^** |  |  |  |  |
| Public Health Wales (PHW) |  |  |  | **x** |  |
| Qresearch |  |  | **x** |  |  |
| ResearchOne |  | **x** |  |  |  |
| Royal College of Emergency Medicine, multiple audits |  |  | **x** |  |  |
| Royal College of General Practitioners (RCGP) Research and Surveillance Centre | **x** |  | **x** |  |  |
| Secure Anonymised Information Linkage (SAIL) ^6^  <https://saildatabank.com/> | **x** | **x** |  |  |  |
| Sentinel Stroke National Audit programme (SSNAP) |  |  |  |  | **x** |
| Serious Hazards of Transfusion (SHOT) |  |  |  |  | **x** |
| Society for Acute Medicine's Benchmarking Audit (SAMBA) |  |  | **x** |  |  |
| The Health Improvement Network (THIN) |  |  | **x** |  |  |
| Trauma Audit and Research Network - Major Trauma Audit (TARN) |  | **x** |  |  |  |
| UK Cystic Fibrosis Registry (UKCFR) |  | **x** |  |  |  |
| UK Parkinson’s Audit |  |  |  |  | **x** |
| UK Renal Registry (UKRR) |  | **x** |  |  |  |

Footnote: ^1^Since 2015, ^2^Although registry staff informed us that no trials had accessed the data, we identified one or more trials, ^3^Since 2013, ^4^Since 2010, ^5^Since 2014, ^6^website given of the top 4 giving data to clinical trials in 2013-2018
