# Supplementary material for: Access to routinely collected health data for clinical trials – review of successful data requests to UK registries
Source: Trials. 2020 May 12;21:398. doi: 10.1186/s13063-020-04329-8 (PMC7218527; doi:10.1186/s13063-020-04329-8)
Supplement: Supplementary file 2 — Additional file 2: Table S2. Linkage identifier combinations by frequency of use (detailed extraction 2017–2018). [file 13063_2020_4329_MOESM2_ESM.docx]

**Table S2. Linkage identifier combinations by frequency of use (detailed extraction 2017-2018)**

| **Frequency** | **NHS number** | **Name** | **Initials** | **Date of birth** | **Post code** | **Gender** | **Unclear** | **Not applicable** |
| --- | --- | --- | --- | --- | --- | --- | --- | --- |
| 62 |  |  |  |  |  |  | **X** |  |
| 20 | **X** | **X** |  | **X** |  |  |  |  |
| 9 | **X** |  |  | **X** | **X** |  |  |  |
| 8 | **X** |  |  | **X** | **X** | **X** |  |  |
| 7 | **X** | **X** |  | **X** | **X** | **X** |  |  |
| 7 | **X** |  |  | **X** | **X** |  |  |  |
| 6 | **X** |  |  | **X** |  |  |  |  |
| 6 | **X** |  |  |  |  |  |  |  |
| 3 |  |  |  |  |  |  |  | **X** |
| 1 |  | **X** |  | **X** | **X** |  |  |  |
| 1 | **X** |  |  | **X** |  | **X** |  |  |
| 1 | **X** |  | **X** | **X** |  |  |  |  |
| 1 | **X** | **X** |  |  | **X** |  |  |  |
| 1 | **X** | **X** |  | **X** |  | **X** |  |  |
| 1 | **X** | **X** |  |  |  |  |  |  |

Caption: This table displays the frequency with which various combinations of identifiers were used for linkage in the registries accessed. X = identifier contributed to combination
