# Supplementary material for: Access to routinely collected health data for clinical trials – review of successful data requests to UK registries
Source: Trials. 2020 May 12;21:398. doi: 10.1186/s13063-020-04329-8 (PMC7218527; doi:10.1186/s13063-020-04329-8)
Supplement: Supplementary file 3 — Additional file 3: Table S3. Data use combinations by frequency of use, among trials using data for the primary report (n = 74). [file 13063_2020_4329_MOESM3_ESM.docx]

**Table S3. Data use combinations by frequency of use, among trials using data for the primary report (n=74)**

|  | Data use category | | | | | | | |
| --- | --- | --- | --- | --- | --- | --- | --- | --- |
| Frequency | 1 | 2 | 3 | 4 | 5 | 6 | 7 | Unclear |
| 16 | X |  |  |  |  |  |  |  |
| 10 |  |  |  |  |  |  |  | X |
| 8 |  |  |  | X |  |  |  |  |
| 7 | X |  |  |  |  | X |  |  |
| 6 |  |  |  |  |  |  | X |  |
| 6 |  | X |  |  |  |  |  |  |
| 5 |  |  | X | X |  |  |  |  |
| 4 |  | X |  | X | X |  |  |  |
| 4 |  |  | X | X |  | X |  |  |
| 3 |  |  |  |  | X | X |  |  |
| 3 |  | X | X |  |  |  |  |  |
| 3 | X |  |  | X |  |  |  |  |
| 3 | X |  | X |  |  |  |  |  |
| 2 | X |  | X | X |  | X |  |  |
| 2 | X |  |  |  | X | X |  |  |
| 2 | X | X |  |  |  |  |  |  |
| 2 | X | X | X |  | X | X |  |  |
| 2 |  | X |  |  |  | X |  |  |
| 2 |  | X | X |  | X | X |  |  |
| 2 |  | X | X |  | X |  |  |  |
| 2 |  | X | X | X |  | X |  |  |
| 1 | X | X |  | X | X |  |  |  |
| 1 |  | X |  |  |  |  | X |  |
| 1 |  | X |  | X |  | X |  |  |
| 1 |  | X |  | X |  |  |  |  |
| 1 | X | X | X | X |  |  |  |  |
| 1 |  |  | X |  |  |  | X |  |
| 1 |  |  | X |  |  | X |  |  |
| 1 | X |  |  | X |  |  | X |  |
| 1 | X |  |  | X |  | X |  |  |
| 1 | X | X |  |  |  | X |  |  |
| 1 |  |  | X |  |  |  |  |  |
| 1 | X |  |  |  | X |  |  |  |
| 1 |  | X |  |  | X | X | X |  |
| 1 | X | X | X |  |  |  |  |  |
| 1 |  | X | X |  |  |  | X |  |
| 1 | X |  | X |  | X |  |  |  |
