# Supplementary material for: Access to routinely collected health data for clinical trials – review of successful data requests to UK registries
Source: Trials. 2020 May 12;21:398. doi: 10.1186/s13063-020-04329-8 (PMC7218527; doi:10.1186/s13063-020-04329-8)
Supplement: Supplementary file 4 — Additional file 4: Appendix 1. Additional information about data collection. [file 13063_2020_4329_MOESM4_ESM.docx]

**Appendix 1: Additional information about data collection**

- The data collection process drew on all possible sources of information, not just the purpose statements within the release registers. Wherever possible, information was gleaned from available sources: trial registration pages, protocols, publications, statistical analysis plans and trial websites, including privacy statements which often detailed the nature of any planned record linkages.
- We employed duplicate screening and data extraction in efforts to obtain all relevant trials and reduce errors.
- RCTs may not have used the datasets they accessed, nor used them in the way described in protocols.
- The organisation was captured as that requesting the RCHD and may not reflect the main trial team involved, for example in the case of collaborating institutions applying for RCHD for economic analyses.
- In cases where trials were described as extensions or long-term follow-up of a trial, data were also captured for the trial, for example, the primary outcome was considered that of the primary report. In cases where it was unclear whether data was requested for the primary, or long-term follow-up, the latter was defined as requests for data points occurring after that of the primary analysis as reported in the primary results paper.
- Cluster and stepped-wedge studies were captured as cluster randomised trials
- We captured whether a registered CTU was involved in the conduct of the trial, and searched through the individual CTU websites to cross-check.
- The actual sample size of the trial was captured for trials which had completed recruitment, and for ongoing studies the target sample size was collected. For trials with multiple randomisations, the first randomisation was captured only.
- Trials evaluating an intervention for secondary prevention, for example aspirin to prevent recurrent stroke, were classed as treatment. Secondary care was defined as a trial where one or more interventions were administered in a hospital.
- Drug trials were not necessarily CTIMPs, but any trial evaluating at least one medicinal product, even if for a licensed purpose.
- When trials were published and the publication included, or was considered to include, RCHD, we captured the journal. In cases where multiple publications existed, we captured the journal of the highest profile.
- The name of the trial was not available in all cases, for example in release registries where only an application title was available. Where necessary, descriptions provided (including study methodology and research institution) were matched to identify the trial.
- Some assumptions were made about the RCHD provider used where necessary, for example, hospital episode statistics were presumed to be released by NHS Digital when the registry was not provided or unclear. Releases from predecessor organisations or organisations that have changed name over time, were captured as the most recent organisation name (e.g. Health and Social Care Information Centre (HSCIC) releases were captured as NHS Digital). Similarly, mortality releases from ONS are captured under NHS Digital as it was often unclear which provider had been used, and as this mortality data is now only available from NHS Digital. Additionally, Scottish mortality data was captured as being released by the Information Statistics Division Scotland, however it is known that for at least one trial these data have a legacy contract to flow to NHS digital who then provides the data to the trial team. RCHD used was determined from release registries or results publications, and not from protocols, as not all studies planning to access RCHD may have done so.
- Although many trial privacy statements mentioned record linkage with a number of data providers, it became clear that in many cases these applications had not yet been made or approved. Therefore, we did not rely on this information to inform the RCHD used, and confirmation was required either from the data provider or within trial publications.
- In calculating the total number of RCHD sources, organisations with multiple similar audits or datasets, were grouped into one source.
- We elected not to contact research teams to clarify results as we aimed for these results to reflect information available in the public domain; however this may have reduced some uncertainty in the data collection.
- The review aimed to capture all studies accessing RCHD since 2013, however the release registers for some sources were only initiated more recently. Therefore trials accessing data between 2013 and 2015 may have been missed.
